# Fundamentals of Scholarly Peer Review: A Workshop for Health Professions Educators on Practicing Scholarly Citizenship

**DOI:** 10.15766/mep_2374-8265.11174

**Published:** 2021-08-02

**Authors:** David P. Way, S. Beth Bierer, Anna T. Cianciolo, Larry Gruppen, Janet M. Riddle, Brian Mavis

**Affiliations:** 1 Senior Education Research Associate, Department of Emergency Medicine, The Ohio State University College of Medicine; 2 Associate Professor, Cleveland Clinic Lerner College of Medicine, Case Western Reserve University School of Medicine; 3 Associate Professor, Department of Medical Education, Southern Illinois University School of Medicine; 4 Professor of Learning Health Sciences and Director of the Master of Health Professions Education Program, University of Michigan Medical School; 5 Research Assistant Professor and Director of Faculty Development, University of Illinois College of Medicine at Chicago; 6 Professor, Medical Education Research and Development, Michigan State University College of Human Medicine

**Keywords:** Faculty Development, Educational Personnel, Peer Review, Research Ethics, Publishing/Scholarship

## Abstract

**Introduction:**

Education scholarship requires peer reviewers. For novice scholars, reviewing is an important developmental activity that cultivates deeper participation in the scholarship community. Yet getting started with reviewing is challenging for those not involved with the educational scholarship community. Beyond scientific expertise, reviewers must have a mentoring mindset, skills in providing constructive feedback, and knowledge of common ethical challenges associated with producing and evaluating scholarship.

**Methods:**

Our workshop introduced novice health professions educators to peer reviewing. It included four stimulus presentations about the peer reviewer's mindset and skills, followed by reinforcement activities. The workshop could be adapted to variously sized groups. An 8:1 ratio of participants to facilitators was ideal for activities. Topics covered included considerations before accepting an invitation, the review process, the good citizen approach to reviewing, and reviewer ethics. The session concluded with suggestions for continued development of peer reviewer competencies. The workshop was evaluated using a custom survey.

**Results:**

Throughout 2019 and early 2020, 58 health professions educators and trainees participated in the workshop across varied venues. Evaluations were obtained from 33 participants (57%). Nearly all rated the workshop as high quality and valuable to peer reviewer preparation. Most (26 of 33; 75%) gained confidence about their qualifications to serve as reviewers. Eighty percent (28 of 33) believed they could recognize ethical dilemmas.

**Discussion:**

This workshop provided a springboard for peer reviewing health professions education scholarship. Participants generally praised the experience for introducing them to the world of peer review and preparing them for it.

## Educational Objectives

At the end of this workshop, participants will be able to:

1. Describe the role of peer reviewer as peer mentor.
2. Summarize how a growth mindset and awareness of social and ethical obligations to the health professions education community contribute to the peer reviewer's effectiveness.
3. Write constructive statements that contribute to improving the presentation of scholarly work.
4. Explain common examples of ethics violations during the peer review of scholarly work.

## Introduction

Scholarly scientific dissemination occurs through grant proposals, conference presentations, and journal articles. This enterprise relies heavily on peer review to refine scholarship by assessing its quality and fit to the targeted venue. Quality peer reviewers contribute to improving scholarly work and enhancing its value to consumers.^1^ Ultimately, peer review of scholarly work advances science.

Within and across professional organizations, members form communities of practice (CoPs) through which they develop knowledge and mutual commitment to serving the community.^2,3^ Like a living organism, to survive and thrive, CoPs must continually welcome new members and develop them as part of the community through teaching and mentoring.^4^

New members to the health professions education scholarly CoP come from various backgrounds and include veteran scientists new to educational roles, new members of the academic faculty, and individuals who are planning future careers in academics. For newcomers (novices) who come from disciplines or professions outside of the behavioral and social sciences, peer reviewing in education can be intimidating.^1^ Yet reviewing is an important developmental activity that contributes to establishing one's place within a scholarly CoP.

Peer reviewing offers a gateway for novices into the health professions education scholarly CoP and provides a window into the world of education scholarship. Group and mentored peer reviews help novices learn and practice the critical analytic skills required of good peer reviewers.^5^ Through participation in peer reviewing, novices see how others engage in scholarly activities, apply new methods, and harness resources from unfamiliar professions and disciplines. Novices also learn the rules and guidelines for scientific inquiry and scholarly dissemination shared among community members.^6–11^ Guidelines help novices learn to conduct reviews in a responsible manner, which requires awareness of the reviewer's role in monitoring the review process and the ethical challenges associated with serving as a reviewer.^12^ Peer review not only stimulates new ideas but gives novices a foundation on which to build their own scholarly work, as well as the added benefits of staying current with the literature and learning more deeply about particular subject matter.^13^

While granting agencies, conference planners, and journal editors value scholarly expertise, one need not be an expert in all aspects of the work to serve effectively as a peer reviewer. Individual peer reviewers contribute to the collective critique of a scholarly product, so they need not fear being insufficiently qualified in some aspect of the work being evaluated (including statistics). For this reason, it is important to help novice scholars discover their role as a peer mentor to authors.^14^ This mindset facilitates the provision of constructive, formative feedback with the goal of improving the scholarly product in the future.

Many journal and publisher websites offer tutorials, checklists, and manuals on how to review conference abstracts, manuscripts, or grant proposals.^6,7,9,10,15^ In fact, even *MedEdPORTAL* has published an online module with detailed, technical how-to advice on manuscript reviewing.^16^ However, these resources are designed for intermediate and advanced practitioners of educational scholarship. Because traditional resources are comprehensive, prescriptive, and technical in nature, their format can be intimidating to newcomers or novice scholars who are unsure of their place within a scholarly CoP. As an alternative to solitary reviewer development methods, our workshop is designed to provide a practical, guided experience to welcome novice health professions education scholars into the world of peer reviewing from a developmental perspective. Our workshop offers participation in exercises to try out new skills and discusses the roles and responsibilities of peer reviewers, with the goal of getting novices started in the practice. By inviting novices to the scholarly CoP and starting them on the path to becoming active peer reviewers, we aim to sustain the scholarly scientific dissemination enterprise and build a vibrant community of education scholars committed to serving.

## Methods

This workshop was designed for individuals new to or planning on academic careers in health sciences education who wanted to increase their participation in educational scholarship. The examples and cases were primarily derived from medical education at the UME and GME levels, but the learning objectives and teaching concepts are appropriate for any novice health professions educator. Beginning with straightforward yet important concepts for peer reviewing, the workshop prepared novices to see themselves as peer reviewers and offered initial skill development. Beyond community service, we emphasized the benefits peer reviewing provides for those pursuing academic careers. We provided suggestions for complementary developmental activities, including a list of additional skills and techniques, a compendium of resources, and additional training activities.

Collectively, we, the workshop authors, have extensive experience as medical education scholars. Medical education journals have recognized all six of us for the exceptional quality of our own peer reviews. All of us served as the original presenters and facilitators for workshop 1. Subsequent workshops were presented by individual authors with facilitators from their own institutions.

We discovered that the setting for this workshop could be an in-person classroom, synchronous internet platform, or a hybrid version of the two. However, minimal requirements for any of these settings included configuration flexibility to accommodate both large- and small-group activities, tabletop writing surfaces, and breakout room capabilities. When using internet instruction, workshop organizers will need to ensure stable, reliable internet connectivity and make certain that hard copies of the handout are delivered to participants in advance of the session. We have conducted the workshop without the PowerPoint slides (Appendix A); however, because we believe that the visuals enhance the experience for participants, we recommend that the slides be incorporated into the workshop. We also suggest that qualified workshop presenters have experience in submitting an education-related manuscript or grant and be an experienced peer reviewer or editorial board member for a health sciences education journal. We sought institutional review board (IRB) approval at the three institutions where evaluation data were gathered. All three declared that the project did not fall under the purview of the IRB as human subjects research: Cleveland Clinic Foundation (letter dated August 11, 2019), The Ohio State University (reference 2019E0745), and Southern Illinois University (reference 018624).

### Brief Workshop Agenda

• Introduction (Appendix A, PowerPoint slides 1–3; 5 minutes): This portion was whole-group instruction and ended with the formation of small groups for activity 1.
• Activity 1—reflection on skills, interest, and readiness to be a peer reviewer (Appendix A, PowerPoint slides 4–6; Appendix B; Appendix C, handout pp. 1–2; Appendix D, facilitator guide p. 1; 20 minutes): The activity was performed in small groups of seven to eight participants and a facilitator.
• Activity 2—evaluating helpfulness of peer reviewer's comments (Appendix A, PowerPoint slides 5–7; Appendix C, handout p. 3; Appendix D, facilitator guide p. 2; 25 minutes): Participants were provided with a brief overview of the review process and the proposed mindset of a peer reviewer-as-mentor. This was followed by a small-group activity that reinforced the peer reviewer-as-mentor concept.
• Activity 3—practice reviewing a scholarly sample and writing feedback comments (Appendix A, PowerPoint slides 8–9; Appendix C, handout p. 4; Appendix D, facilitator guide p. 3; 30 minutes): This small-group activity provided participants with actual guided practice in reviewing a scholarly product.
• Activity 4—scholarship case studies, reflection on research and reviewer ethics (Appendix A, PowerPoint slides 10–11; Appendix C, handout p. 5; Appendix D, facilitator guide p. 4; 20 minutes): In large group, the session leader defined terms related to research and reviewer ethics. In facilitated small groups, participants reviewed cases and attempted to identify the ethics violated in each case.
• Workshop wrap-up (Appendix A, PowerPoint slide 12; Appendix C, handout pp. 6–7; 15 minutes): The session wrap-up was designed to help participants plan a course of study to continue to improve their peer review skills.
• Evaluation (Appendix E, workshop evaluation instrument).

### Workshop Progression

#### Workshop introduction

We introduced the workshop using Appendix A, slides 1–3. The introduction was intended to promote the concept of constructive peer review. Workshop facilitators described and gave examples of a growth mindset, a requirement for helping workshop participants view their peer reviewer role as a mentor to the authors whose work they were reviewing.^14^ This mindset was contrasted with peer reviewer as authoritative expert, a fixed mindset seeing the reviewer as a security guard deciding whose work gets accepted and whose does not.

In preparation for the first activity, we gave learners the workshop handout (Appendices B and C), while we guided the facilitators to use the facilitator guide (Appendix D). A second objective of the introduction and of the first activity was to help participants see themselves as sufficiently qualified to tell others about the quality, fit, and potential impact of their work. We suggested that as part of their roles as medical educators, participants were already immersed in the content they taught. We urged them to volunteer as peer reviewers for the venues (grants, conferences, or journals) with which they were most familiar. We suggested that because of their extensive education, they probably already had sufficient background in the basics of teaching, learning, and scientific thinking to write a constructive critique of someone else's scholarly work. We also reminded participants that peer review was a team sport involving two to three peer reviewers, so that any one individual did not have to cover every aspect of a submission.

#### Activity 1—reflection on skills, interest, and readiness to be a peer reviewer

The facilitator provided an overview of the basic qualities of a constructive peer reviewer, emphasizing that general familiarity with the presentation venue (grant, conference, or journal) and with the authors’ target audience was as important as being a content expert. Furthermore, the reviewer should have an interest in the topic and be able to commit the time to provide a quality review.

We directed the participants to form small groups. The ideal small-group configuration to balance individual participation across the group was eight participants per facilitator.^17,18^ Small-group facilitators instructed participants to complete section 1.1 on worksheet p. 1 and section 1.2 on worksheet p. 2 (Appendix C), which constituted a brief profile of their reviewer qualifications. Once participants finished their profile, the facilitator asked them to share one topic of interest from section 1.1 and one skill they self-rated as a 3 or better from section 1.2.

Finally, we asked participants to review section 1.3 on p. 2 of the worksheet (Appendix C), which presented them with an invitation to review a simulated manuscript. Participants were asked to read the abstract and decide whether they would accept the invitation. Once everyone had generated an answer, the facilitator asked each participant to state their decision and rationale.

#### Activity 2—evaluating helpfulness of peer reviewer's comments

The facilitator briefly explained the peer review process and what happened after reviewers submitted their comments. One purpose of this overview was to show participants that even though they typically communicated with one decision-maker, they were part of a team; each reviewer brought complementary skills to the task. This helped the participants understand that even if they were not an expert in research methods or theoretical content, they could represent a typical consumer of the scholarly work.

The facilitator then defined the concept of peer reviewing as mentoring,^14^ which involved providing constructive feedback to the authors and guidance on how the submission's contribution could be improved. We also promoted the good citizen approach to peer reviewing, which was to be respectful but authoritative (supportive but definitive) in identifying strengths and weaknesses of the submission. Just as effective supervisors provide high-quality feedback to their clinical learners, peer reviewers should provide specific, detailed, actionable feedback with concrete examples and specific recommendations for improvement.^19^ If there was a fatal flaw with the piece, it was the reviewer's obligation to provide guidance on how the authors could revise their work. If the reviewer was able to recognize a flaw but unable to provide guidance, they were instructed to bring the flaw to the editor's attention.

Following this discussion, we directed participants to small groups and completed an activity designed to reinforce the concept of peer reviewing as mentoring. The assumption motivating this exercise was that being able to recognize and remediate helpful comments would make participants better able to craft helpful comments themselves. Participants were asked to evaluate how helpful selected excerpts of actual reviewer comments were to authors and decision-makers (handout p. 3, Appendix C). After participants had completed their ratings, facilitators led a brief discussion to see whether participants agreed upon the ratings they had assigned. If there was time, participants were asked to try to improve the comments considered marginally helpful.

#### Activity 3—practice reviewing a scholarly sample and writing feedback comments

In activity 3, facilitators guided small groups of participants through the review of a one-page research abstract. We asked the participants to generate a list of the abstract's strengths and weaknesses and to provide their overall impressions. Optionally, they also provided recommendations for improvement.

The facilitator offered the small groups the opportunity to report their reviews to the whole group. The questions on slide 9 (Appendix A) provided structure for this debriefing. The leader pointed the participants to these questions when they needed prompts to promote participation. Just showing the slide provided guidance to help reporters frame their comments. Activity 3 concluded with a vote among the audience members as to what the editor's decision should be regarding the fate of this abstract.

#### Activity 4—scholarship case studies, reflection on research and reviewer ethics

The facilitator introduced this activity with additional reviewer qualifications, including that reviewers must have a basic understanding of scholarly ethics regarding not only author conduct but also the conduct of everyone else involved in scholarly dissemination. This meant that reviewers were charged with the task of judging whether the authors had followed scientific principles and upheld scholarly ethics and also with ensuring that their own behavior was ethical.

Together, each small group reviewed the cases found on handout p. 5 (Appendix C). After giving participants time to read the cases, the facilitator led a discussion about each one, asking, “Does this case make you feel uncomfortable? If so, why?” Next, the facilitator provided descriptions of the seven most common ethics violations that we had identified in our work as authors, editors, and reviewers. The facilitator questioned participants on their understanding, then asked them to match the cases to these descriptions to develop a vocabulary for describing the ethical problems they identified. When time permitted, the audience was asked to provide examples of ethics problems not illustrated by the cases.

#### Workshop wrap-up

The wrap-up reinforced key concepts (listed on slide 12, Appendix A) and used p. 6 of the handout (Appendix C) to show how these learning objectives fit into a larger body of literature on peer reviewing. The facilitator took general questions and then called participants’ attention to the additional resources listed on p. 7 of the handout (Appendix C). The facilitator pointed out that the additional resources had been selected to help participants map out a plan for continued development as a constructive peer reviewer.

#### Evaluation

We designed a workshop evaluation form (Appendix E) to gather participants’ formative feedback. The first part of the form contained items assessing workshop objectives, such as whether participants could describe the role of the reviewer, discuss the growth mindset required of a mentoring peer reviewer, write constructive statements, and explain issues of scholarly ethics. The second set of items asked participants to rate the quality and value of the workshop's components. Finally, we asked participants to state the single best thing they had learned from the workshop and to offer suggestions for how the workshop could be improved. All rating-scale evaluation items were analyzed with IBM-SPSS Statistics for Windows and reported as descriptive statistics. Comments derived from the open-ended survey question were grouped into categories through simple thematic analysis.

## Results

From April 2019 through February 2020, we piloted the workshop five times across a variety of settings with diverse learners. The workshop was easily adapted to learner needs, number of available facilitators, and conditions at each site. In one instance, the workshop was delivered face-to-face in a conference room and synchronously online with learners off-site.

The workshop was first conducted at the AAMC's Central Group on Educational Affairs (CGEA) 2019 Spring Conference. All six of us presented the workshop and facilitated small-group discussions. The session took place in a hotel conference room equipped with nine to 10 round-top conference tables and standard presentation equipment (screen, projector, and lectern). This workshop was attended by 19 medical educators from throughout the central region of the US. While we did not conduct or receive evaluations for this workshop, anecdotal evidence of its effectiveness was received in the form of enthusiastic participation by the conference attendees, their personal feedback after the session, and their attendance at a panel discussion by some of us on the same topic later in the day.

We carried out the remaining workshops at our own institutions (The Ohio State University, Cleveland Clinic Foundation, and Southern Illinois University).

• We delivered workshop 2 to nine junior emergency medicine faculty, fellows, and residents interested in developing skills in medical education scholarship. This session took place in a small conference room with seating around one large conference table and standard presentation equipment.
• We implemented workshop 3 in a classroom with seven participants enrolled in a longitudinal faculty development certificate program called Essentials of Teaching. Participants included both clinical educators and education coordinators.
• A central faculty development unit marketed and organized workshop 4 across an entire medical school. Subsequently, this workshop was delivered synchronously across two campuses: one in person and one off-site. The workshop attracted a diverse audience of 12, including education administrators, residency program leaders, foreign exchange students, and individuals considered underrepresented in medicine. Both sites were set up in classrooms outfitted with videoconferencing capability over the internet.
• A psychiatry department, which included 11 faculty and residents, sponsored workshop 5. This session was modified to take place in a faculty member's home without audiovisual or lecture equipment.

To date, 58 people have participated in the workshop, 33 (57%) of whom completed most of the evaluation survey. One-third of the participants (19 of 58; 33%) were already in educator roles. Other participants (15 of 58; 26%) included trainees (fellows, residents, and medical students) interested in academic careers. Unfortunately, we did not ask the roles of CGEA conference attendees but assumed that most had a role in medical education.

Besides differences in settings, primary differences between workshops had to do with the size and composition of the audience. Attendees at workshop 1 were primarily medical educators seeking faculty development opportunities to advance their careers. Attendees at the remaining workshops were a mix of junior faculty, education administrators, and trainees (either medical students, residents, or fellows). Furthermore, the size of the groups for workshop 2 (*n* = 9) and workshop 3 (*n* = 7) made it possible for participants to work as one group for the entire session.

### Evaluation Related to Objectives

No differences across sites were observed, so evaluation results were aggregated. In response to questions regarding the workshop objectives, participants unanimously agreed that peer reviewers played a role in improving scholarship through their constructive comments. Participants also agreed with the principle of reflecting on whether they had the time and skills before committing to review (Table 1). More than 80% of the participants felt they would be able to recognize issues related to scholarly ethics during the peer review process. Slightly more than three-fourths of the participants thought that they could be a good peer reviewer.

**Table 1.**
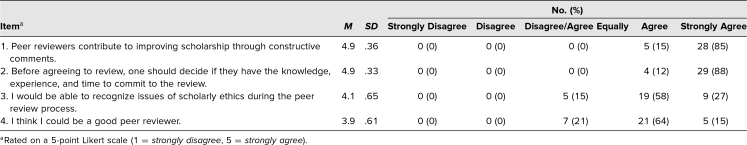
Evaluation Responses From 33 Workshop Participants

### Evaluation of Value

Most participants (90% or more) found the activities to be of considerable or great value to their preparation to become a peer reviewer (Table 2). Exceptions were the introduction and the wrap-up. Those who thought the introduction was of moderate value suggested that this time should be allocated to activities. Those who rated the wrap-up low in value said that this activity had been cut from their session. One workshop group suggested that the introduction and the wrap-up contributed to the session running too long, while another suggested that the time allocated for the workshop needed to be extended.

**Table 2.**
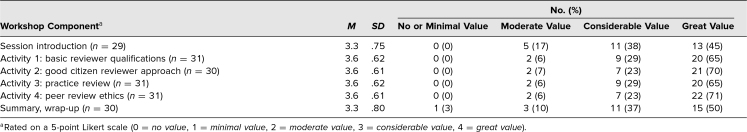
Evaluation Responses Rating the Workshop's Value in Preparing Participants to Be a Peer Reviewer

### Evaluation of Quality

All participants rated the quality of two of the six workshop activities as good or excellent (Table 3). Participants rated the other activities lower, in part due to technical difficulties with delivering the workshop via videoconference technology (e.g., web-based interface issues and late arrival of handouts, materials arriving midway through the second activity). One individual scored the quality of four of the activities as satisfactory or fair because the titles on the evaluation form did not precisely describe the activities. Those who rated the introduction and wrap-up activities as moderate included participants who had experienced a shortened version of these activities or had not experienced them at all.

**Table 3.**
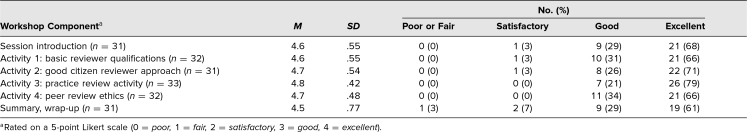
Evaluation Responses Rating the Quality of the Workshop Instruction

### Participant Feedback

Workshop participants took away a variety of important concepts from the workshop. This finding was probably attributable to variability in prior knowledge, backgrounds, and experience that participants brought to the workshop (see the Figure). Many participants became aware that they could begin contributing as a peer reviewer and/or suggested that they understood that peer reviewing was an important part of academic citizenship. Others valued the ethics activity, suggesting that the material covered by this activity, including the concepts related to salami slicing, obtaining IRB approval, and rules governing authorship, was new and unfamiliar. Finally, participants suggested that they understood that peer reviewing involved having a growth mindset through the provision of constructive feedback.

**Figure.**
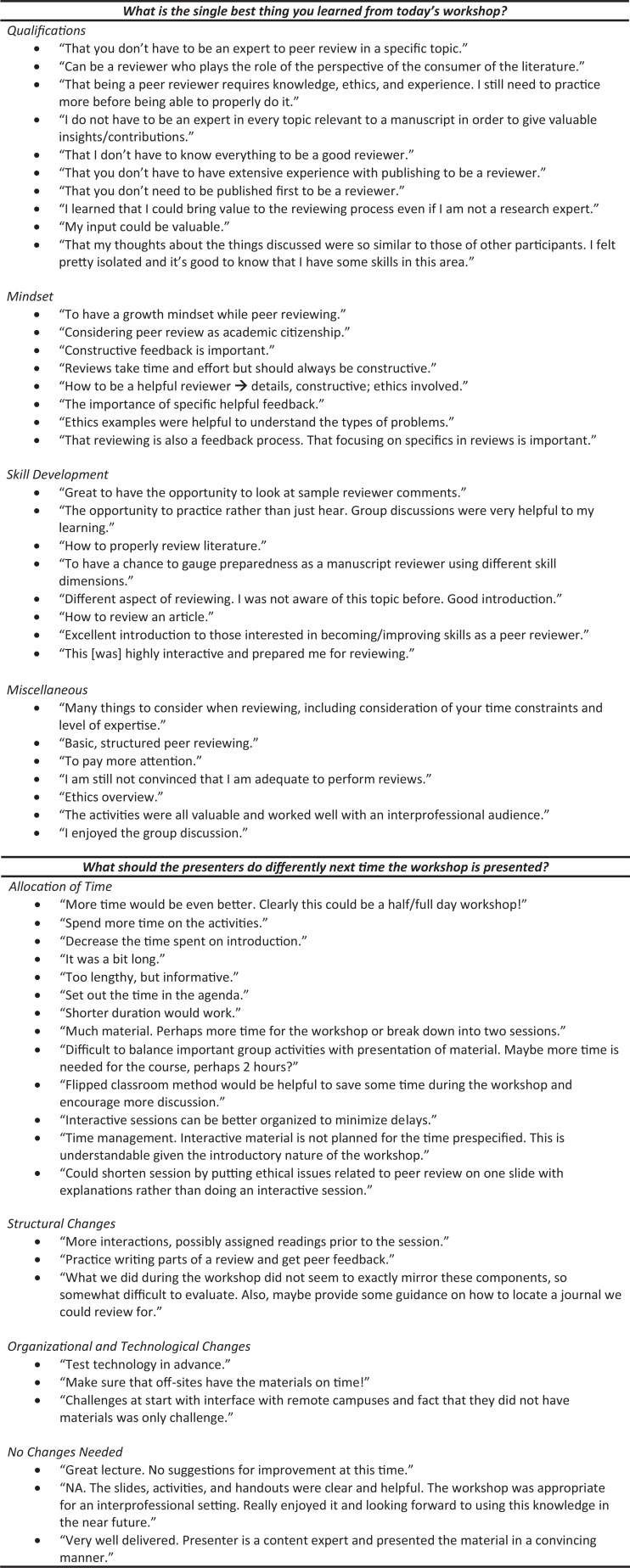
Participant reactions to open-ended evaluation questions. These verbatim responses have been grouped into thematic categories for each question using simple thematic analysis.

The most consistent theme coming from the recommendations for improvement centered on dedicating more time to the activities. Those who suggested lengthening the time of the workshop proposed 2 hours or more. One individual suggested the provision of more specific guidance on identifying journals for which participants might review. A few participants from one of the five sessions complained about the workshop running too long.

### Evaluation Related to Delivery

Our first foray into delivering the workshop through distance learning did not go well. Delivery to the off-campus site was delayed due to a poor internet connection. Additionally, logistical challenges with the off-campus site prevented the handouts from reaching the participants on time. Evaluations from distance learners (*n* = 3) offered numerous suggestions for ways to improve the workshop. These individuals suggested that the combination of outdated videoconferencing technology and lack of immediate access to the workshop materials (handouts) made them feel like they did not get the full workshop experience.

## Discussion

The workshop met the interim objectives of engaging novice medical education scholars in simple and practical activities that piqued their interest in becoming peer reviewers. Self-efficacy for peer reviewing was improved, with most participants believing that they could become good peer reviewers. Experiences with piloting the session in different settings provided feedback needed to improve and tailor the workshop. We found the workshop to be adaptable to a variety of settings and learners. While the structure and nature of the activities could easily be adapted to other professions, we specifically designed this workshop for health professions educators. Accordingly, we changed the title of the workshop to communicate the target audience.

### Reflections and Lessons Learned

Our experience with piloting the workshop taught us that the best setup for delivery is a room with flexible seating that can be configured for both lecture and small-group work. At a minimum, participants need a surface to write on. When conducting the workshop through distance learning, we found it best to arrange for delivery of hard copies of the instructional materials to participants in advance of the event. When conducting the workshop over distance, we highly recommend testing of videoconferencing or internet meeting platforms for connectivity and stability in advance of the session.

Most of the challenges we experienced with distance learning had to do with outdated equipment or lack of coordination across sites. More recently, online meeting platforms with individual connections such as Zoom, Teams, or Go-to-Meeting have become more ubiquitous, making it simpler to conduct this workshop over the internet. Out of necessity during the COVID-19 pandemic, individual educators have become more adept at using online meeting platforms and their features, such as polling and small-group breakout rooms, for promoting active learning. We believe that as long as participants have access to the printed copies of the Appendix C handout in advance, the workshop can be easily and effectively adapted to synchronous internet delivery through the use of online meeting platforms with individual connections.

Most modifications we made after the pilot events have been designed to guide participants more efficiently through the activities, by spending less time on explanations and more time on activities. We added resources to the Appendix C handout so that once individuals get started with peer reviewing, they can use these resources to hone their skills over time. These resources include the AAMC's *Annotated Bibliography of Journals for Educational Scholarship,*^20^ an article on how to perform group or mentored peer reviews,^5^ and an article about engaging in journal clubs to practice critical review of scholarly products.^21^ Also included in the handout are suggested activities to help participants engage in peer reviewing after completing the workshop.

The original activity 1 included a reference to the acronym IMRAD, used to represent the standard structure (introduction, methods, results, and discussion) for formatting abstracts or manuscripts related to educational research. Despite having experience with medical education research, many early participants lacked familiarity with this acronym. Accordingly, we decided to drop it from activity 1; knowing the IMRAD acronym is not considered a necessary qualification for becoming a peer reviewer, especially since numerous scholarly products, such as descriptions of innovations, do not fit the IMRAD structure. We also reformatted the Appendix C handout to reflect that activity 1 is a compilation of three subactivities.

We retitled each of the activities and the wrap-up to be more descriptive of what participants experience. We made these changes to all materials participants receive: the Appendix C handout, Appendix E evaluation, and Appendix A PowerPoint slides. Additionally, we clarified instructions for each activity and added more space for participant responses on the Appendix C handout.

Finally, we reformatted our original lesson plan into a more comprehensive facilitator's guide to assist others with anticipating participants’ questions and concerns and to promote constructive small-group discussions. The revised Appendix D facilitator's guide incorporates questions that we experienced during the first five pilot workshops and includes suggestions for how to prompt groups that struggle with the tasks required by the activities.

### Limitations

Our evidence of workshop effectiveness is limited to participant self-assessment of learning objective achievement and participant opinions about the value and quality of workshop activities. To obtain a more objective measure of workshop impact, evidence of behavior change would be more desirable. We suggest that future evaluations of this instructional intervention include the tracking of participants over time to gauge their involvement in peer review. Additional evaluation research might also attempt to determine whether a 2-hour workshop is sufficient or whether actual behavior change requires further supplemental interventions.

We must acknowledge the medical education lens through which we developed this workshop. Despite our hope that the workshop could be useful to all health professions educators, our perspective may be distracting to health educators from other health professions.

### Summary

This workshop not only helped novice health professions education scholars see themselves as qualified reviewers, it also framed the peer review process as essential to improving scholarly scientific dissemination. To achieve this aim, peer reviewers were asked to be a mentor and good citizen—to use a growth mindset and provide constructive feedback. Most participants demonstrated receiving this message by their participation in small-group discussions and their evaluation form responses.

The social constructivist learning theory format of the workshop, along with the facilitated small-group discussions and experiential learning activities, was effective in promoting self-efficacy as reviewers. Many of the comments received anecdotally or through the workshop evaluations helped us see that participants felt invited into the health professional educator scholarly CoP.

By disseminating this workshop, we aim to continue growing the health professions education reviewer pool and the quality of peer reviews. In addition to promoting mentored or group peer reviewing and journal clubs to encourage individuals to join the scholarly CoP, we hope that subsequent workshops will be developed to build on the peer reviewer skills we have initiated with this workshop.

## Appendices

Peer Reviewer Training Presentation.pptxHandout Cover.pdfHandout and Activity Worksheets.docxFacilitator Guide.docxEvaluation.docx
*All appendices are peer reviewed as integral parts of the Original Publication.*
