# Supplementary material for: Fundamentals of Scholarly Peer Review: A Workshop for Health Professions Educators on Practicing Scholarly Citizenship
Source: MedEdPORTAL. 2021 Aug 2;17:11174. doi: 10.15766/mep_2374-8265.11174 (PMC8335801; doi:10.15766/mep_2374-8265.11174)
Supplement: Supplementary file 1 — Peer Reviewer Training Presentation.pptxHandout Cover.pdfHandout and Activity Worksheets.docxFacilitator Guide.docxEvaluation.docx [file B. Handout Cover.pdf › B. Handout Cover.pdf]

Fundamentals of Scholarly Peer Review

A Workshop for Health Professions Educators on Practicing Scholarly Citizenship

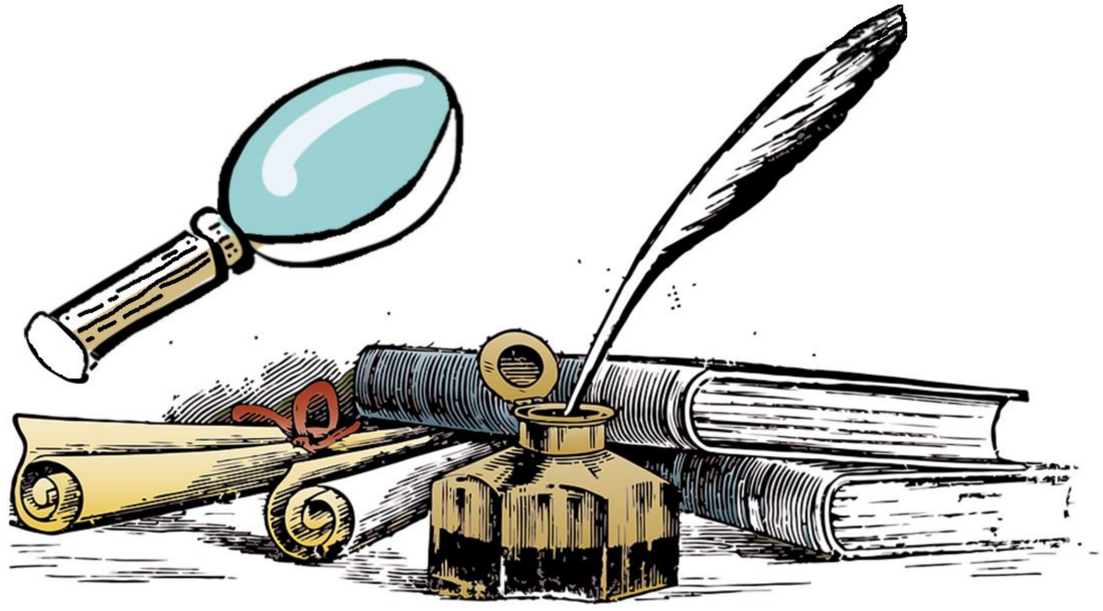

David P. Way, MEd,[1] S. Beth Bierer, PhD,[2] Anna T. Cianciolo, PhD,[3] Larry Gruppen, PhD,[4] Janet Riddle, MD,[5] Brian Mavis, PhD[6]

Ohio State University Department of Emergency Medicine,[1]
Cleveland Clinic Lerner College of Medicine of CWRU,[2]
Southern Illinois University School of Medicine,[3]
University of Michigan Medical School[4],
University of Illinois College of Medicine-Chicago,[5]
Michigan State University College of Human Medicine[6]

Magnifying Glass Clipart image by unknown, retrieved from: http://res.publicdomainfiles.com/pdf_view/60/13534575618789.png on February 1, 2021. Image is in the public domain. Writing, Quill, Books Clipart image by Janet Gooch, retrieved from Pixabay: https://cdn.pixabay.com/photo/2015/11/14/20/26/writing-1043622_960_720.png. Free for commercial use, no attribution required.
