# Supplementary material for: Fundamentals of Scholarly Peer Review: A Workshop for Health Professions Educators on Practicing Scholarly Citizenship
Source: MedEdPORTAL. 2021 Aug 2;17:11174. doi: 10.15766/mep_2374-8265.11174 (PMC8335801; doi:10.15766/mep_2374-8265.11174)
Supplement: Supplementary file 1 — Peer Reviewer Training Presentation.pptxHandout Cover.pdfHandout and Activity Worksheets.docxFacilitator Guide.docxEvaluation.docx [file mep_2374-8265.11174-s001.zip › D. Facilitator Guide.docx]

**Appendix D: Reviewer Training Workshop Facilitator Guide**

**Fundamentals of Scholarly Peer Review: A Workshop for**

**Health Professions Educators Practicing Scholarly Citizenship**

David P. Way, MEd,^1^ S. Beth Bierer, PhD,^2^ Anna T. Cianciolo, PhD,^3^ Larry Gruppen, PhD,^4^

Janet Riddle, MD,^5^ Brian Mavis, PhD^6^

Ohio State University Department of Emergency Medicine,^1^

Cleveland Clinic Lerner College of Medicine of CWRU,^2^ Southern Illinois University School of Medicine,^3^ University of Michigan Medical School ^4^, University of Illinois College of Medicine-Chicago,^5^

Michigan State University College of Human Medicine^6^

**Rationale:**

The dissemination of scholarship requires an army of well-prepared peer reviewers. Recruiting reviewers in medical education research is a challenge since most participants are not trained, nor have opportunities for training. Forming the proper mindset for reviewing education research is a first step to cultivating reviewers as peer mentors. This workshop is designed to provide participants the general mindset and basic skills for becoming peer reviewers for conference abstracts, journals and grants.

**Learning Objectives:**

1.	Describe the role of peer reviewer as peer mentor.

2.	Summarize how a growth mindset and awareness of social and ethical obligations to the health professions education community contribute to the peer reviewer’s effectiveness.

3.	Write constructive statements that contribute to improving the presentation of scholarly work.

4.	Explain common examples of ethics violations during the peer review of scholarly work.

**Session Methods and Format:**

The session includes several “stimulus” presentations about reviewer skills (5 minutes each) that lead into reinforcement activities done in small groups. The small group activities work best if there is no more than an 8:1 ratio of participants to facilitators. Pilot studies of this workshop indicate that the ideal time allocation is 2 hours (120 minutes).

**Facilitator’s Role:**

The role of the facilitator is to guide the small group discussion to ensure that the participants can accomplish the required tasks and that everyone has a positive learning experience. Facilitators should read this guide and the participant handout in advance of the session. They should know the time allocations for each small group activity and be able to recognize when their group is lagging behind and need prompting to get back on schedule. Facilitators should be adept at asking probing questions when the group gets stuck and skilled at interrupting to get the group moving in the right direction.

**Introduction of session**	5 minutes

**Session Leader [Slide 1]** This workshop was borne out of the need to identify and recruit quality peer reviewers for conference submissions, journals and grants. The workshop was also designed to help you, the participant become involved with the medical education scholarship community, a step you will need to take to advance your career.

For this workshop, we go beyond the traditional methods of reviewer development…giving you an article to read, having you review it, then giving you feedback. Instead we provide you with practical guided experiences to get you started.

**Session Leader [Slide 2]** One of the primary goals of this workshop is to emphasize the importance of approaching the task of peer review with a “growth mindset.” In other words, the best reviewers are committed to “helping” the authors, their peers, to improve their work…whether that be a manuscript, a conference abstract or a grant proposal. The goal of the reviewer is not to denigrate or demean the efforts of the authors, but rather to serve as an undercover mentor. Yes, as a reviewer you have the responsibility of contributing to the quality of medical education research that gets published, but you also have the responsibility to nurture those who make the effort to share their scholarly work.

The goal of the reviewer should always be to help make the scholarship better. We do this by preparing ourselves to write constructive feedback about the articles, grants, and conference abstracts that we review. So today’s activities are designed to help you formulate a constructive mindset and get some practice with writing constructive feedback.

**Activity 1**	25 minutes

**Session Leader [Slide 3; Handout pages 1-2]** (3 minutes) To be a good reviewer, one need not be an expert on the topic of the scholarly product. In fact, some of the best reviewers represent the consumers or readership of the scholarly product. So more important than content expertise is a familiarity with the venue of interest (journal, conference, or grant), a basic knowledge about research or scholarly writing, and the interest in time to devote to providing constructive comments about the product to be reviewed.

So before accepting an offer to review a manuscript or abstract, a reviewer must reflect upon whether they have sufficient familiarity with the venue, an interest in the subject, and the time to get the job done.

The purpose of Activity 1 is to develop a brief profile of your reviewer qualifications. We will ask you to share your profile, and then a review invitation will be offered. We will ask you to decide as a group, who among you is best qualified to review the abstract.

**Facilitator [Slide 4; Handout page 1-2]: *Help the session leader arrange the class into small groups, ensuring that there are no more than eight participants in each small group. Once assembled, call your group’s attention to the handout, and ask participants to complete the Activity 1 worksheet parts 1.1 and 1.2 (only). When they are finished with those two sections, they should indicate readiness to move on by putting their writing utensil down. (5 minutes)***

***Please note, that Activity 1 also serves as an ice breaker to introduce individuals to the rest of their group.* *When members are ready, ask them to introduce themselves (name and institution), one topic of interest, and the one skill from the skillset they rated the highest (allocate 30 seconds per member).***

***Call the group’s attention to worksheet part 1.3. The group is collectively playing the role of Dr. Shackleton. Ask them to read the invitation to review the submission, including the abstract. Prompt them to reflect upon their own qualifications (interest, skillset, and time) and determine if they should accept the invitation by circling either “yes” or “no” (5 minutes is allocated for this, however if they are finished quicker, use the extra time for processing this activity).***

***By show of hands, how many would accept this invitation? (Call on 1 person at time to state why they would accept…up to 3 or 4 times). Ask individuals who did not raise their hands why they would not accept the invitation. (Call on 1 or 2). Finally, ask for nominations from the group for the individual you collectively think is most qualified to review this submission. Self-nominations are welcomed. Chose 1 peer-reviewer to present to the whole group with an explanation as to why that individual was selected. (8 minutes)***

**Activity 2: [Slides 5 & 6; Handout page 3]**	(25 minutes)

**Session Leader [Slide 5]** (2 minutes) We want to provide an overview of the peer review process. I am using a journal example however similar processes are experienced for conference submissions and grant proposal reviews. Typically, in the peer review process the scholarly product or submission is screened by a journal editor, a program committee chairman or a grant manager. The editor weighs the merits of the submission, and when they think a submission meets the minimal criteria for that venue, it will be sent out for peer review. Ideally, reviewers are representative of the methods expert, the customer or end user, and a content expert. These individuals conduct their review and return it to the editor for summarizing their work into a report. The important point here is that reviewers actually play different roles or represent different perspectives.

**Session Leader [Slide 6]** (3 minutes) The “good citizen” reviewer approaches the role by providing constructive feedback to the authors, including suggestions for improvement to help move the science forward. The idea is to be respectful, but authoritative (supportive, but definitive) in identifying strengths and weaknesses of the scholarly product, provide detailed actionable feedback and specific recommendations for improvement. If there is a fatal flaw with the submission, the reviewer should provide guidance as to how the authors can replicate their work and get it right the second time.

I like to accomplish these tips by directing my comments to the editor. I imagine that I am writing a letter to the editor and summarizing for him or her what I think the authors’ objectives were. I give my overall impressions by suggesting how I think the work contributes to the literature and whether this will add to that body of knowledge…even on an incremental scale. I often provide references that I think would help the authors. I then provide strengths and suggestions for improvement and an overall recommendation as to what I think should happen next with this submission.

For activity 2 **[Slide 7]**, we have provided you with real excerpts from reviews. In your groups, we ask that you critique and discuss these comments and decide if they are marginally helpful, somewhat helpful, or very helpful. If you have time, try to fix those comments that are marginally helpful.

**Facilitator [Handout page 3]: Activity 2 is on page 3 of the handout. *Ask the participants to provide their rating of each comment in Column A (5 minutes)*.**

***Ask one of the participants to say what their rating of the first comment was and why. Allow the participants to discuss…i.e. What do the rest of you think about that? If there is not unanimous agreement, try to draw out the voice of those who disagree. Urge the group to reach consensus (if possible) then in Column B place the group rating. Continue in this manner, selecting a different participant to start the conversation for each of the reviewer comments. Some of them will go quickly, because they all agree.***

***Since you will have more comments to process than you have participants, it is best to try to ask a participant who did not get to lead the processing of a contested reviewer comment to go a second time.***

***If there is time remaining after you have reached group consensus on all 10 items, have the group go back and “improve” the items that were rated a 1-marginally helpful (15 minutes).***

**Activity 3:** **[Slides 8-9]**	(30 minutes)

**Session Leader [Slide 8; Handout page 4]** (2 minutes). Now we want to take move from recognizing constructive comments to actually generating them yourselves. This activity involves reviewing a short submission, kind of like the type you would receive for a proposed presentation at a conference. Your charge is to peer review the submission and provide 1) a simple overview of what the submission is about, 2) a list of strengths of the submission, 3) a list of weaknesses, and 4) a recommendation to the editor. We will ask that your group report back out to the whole group with your decision and reason behind it.

**Facilitator [Handout page 4]: *Direct the group’s attention to the submission on page 4. Suggest that after reading the whole submission, that they may want to divvy up the task of critiquing different parts of the submission (i.e. break it up by introduction, methods, results, discussion). (This is not necessary, but will be more practical for groups that tend to move more slowly). Ask individuals to take notes that they can compile for a group spokesman to present to the whole group.***

***If you break up the submission into parts, save some time for small group discussion before presenting to the large group. When they have completed the tasks, ask whether their constructive criticism contributes to improving the submission…and/or whether their comments are helpful. (20 minutes).***

**Session Leader [Slide 9; Handout page 4]:** Show slide 9, but do not comment. Allow each of the small groups to present their results (2 minutes each). If time, provide some recognition regarding unique comments and/or comments common among all of the groups.

**Activity 4:** **[Slides 10-11]**	(20 minutes)

**Session Leader** **[Slides 10-11; Handout page 5]** (5 minutes)**:** One of the important competencies a peer reviewer needs is a basic understanding of scholarly ethics, not only from the authorship side, but also the publisher’s and reviewer’s perspective too. Part of the reviewer role is to make judgements about whether the authors of the work they are reviewing have followed scientific principles and upheld scholarly ethics. However, the reviewer must also ensure that their own behavior is ethical. Let’s first define each of these common ethical problems and then see if we can identify them in action.

Define each of the common problems listed on slide 11:

- Salami slicing: The slicing of research that would normally form one cogent or meaningful paper into two or three smaller papers, in the hope of bolstering one’s publication record.
- Self-plagiarism: Reusing or repeating one’s own writing from one publication into another publication without proper citation.
- Literature misrepresentation/plagiarism: Using someone else’s work and representing it as one’s own. OR Mischaracterizing or misrepresenting someone else’s scholarly work.
- No proper IRB process/ permission to conduct human subject’s research: All research involving human subjects must have the approval of an internal review board. Without proper permission from an IRB, one cannot carry out human subject’s research.
- Reviewer/Author confidentiality violation: Many journals enforce what is called blind review, meaning that they do not want the reviewer to know the identity of the authors, and vice versa. If this is the journal’s rules, they should be followed.
- Recognizing conflicts of interest: When a reviewer’s self-interest, like helping a friend get published gets in the way of an objective peer review, there is a conflict of interest. Conversely, if an author wants to improve their chances of getting published, they may game the system by proposing a reviewer who will give them a favorable review.

**Facilitator [Handout page 5]: *Direct the group’s attention to the 5 behaviors exhibited by authors/reviewers on handout page 5. After the group has read these cases, ask the group members to suggest which ethical challenge each case represents. Be sure that if they do not provide a rationale that you probe or stretch the respondent to get them to explain why they think the case fits into that category. Work through all 5 cases. If there is extra time, ask the group to come up with a case that represents the missing category. (15 minutes)***

**Wrap-up:** [Slide 12]	(15 minutes)

**Session Leader** Audience will see a concluding visual that summarizes how to be a good peer reviewer. The facilitator will go through the list of skills that was covered in the workshop, and some additional skills that participants can pursue on their own. They will also receive a handout on where to receive additional information or training on how to be a good reviewer.
