# Supplementary material for: Fundamentals of Scholarly Peer Review: A Workshop for Health Professions Educators on Practicing Scholarly Citizenship
Source: MedEdPORTAL. 2021 Aug 2;17:11174. doi: 10.15766/mep_2374-8265.11174 (PMC8335801; doi:10.15766/mep_2374-8265.11174)
Supplement: Supplementary file 1 — Peer Reviewer Training Presentation.pptxHandout Cover.pdfHandout and Activity Worksheets.docxFacilitator Guide.docxEvaluation.docx [file mep_2374-8265.11174-s001.zip › E. Evaluation.docx]

**Appendix E: Workshop Evaluation**

**Fundamentals of Scholarly Peer Review: A Workshop for Health Professions**

**Educators on Practicing Scholarly Citizenship**

David P. Way, MEd,^1^ S. Beth Bierer, PhD,^2^ Anna T. Cianciolo, PhD,^3^ Larry Gruppen, PhD,^4^

Janet Riddle, MD,^5^ Brian Mavis, PhD^6^

Ohio State University Department of Emergency Medicine,^1^

Cleveland Clinic Lerner College of Medicine of CWRU,^2^ Southern Illinois University School of Medicine,^3^ University of Michigan Medical School ^4^, University of Illinois College of Medicine-Chicago,^5^

Michigan State University College of Human Medicine^6^

**DIRECTIONS**: Please take a few moments to provide formative feedback to the authors for improving

this workshop. Circle the response that best matches your opinion. Use the following key:

**SD= Strongly Disagree; D=Disagree; D/A= = Disagree/Agree Equally; A=Agree; SA= Strongly Agree**

| 1. Peer-reviewers contribute to improving scholarship through constructive comments | **SD** | **D** | **D/A=** | **A** | **SA** |
| --- | --- | --- | --- | --- | --- |
| 2. Before agreeing to review, one should decide if they have the knowledge, experience and time to commit to the review | **SD** | **D** | **D/A=** | **A** | **SA** |
| 3. I think I could be a good peer reviewer | **SD** | **D** | **D/A=** | **A** | **SA** |
| **As a result of participating in this workshop, I am better able to…** |  |  |  |  |  |
| 4. … write constructive statements that contribute to improving the presentation of scholarly work | **SD** | **D** | **D/A=** | **A** | **SA** |
| 5. … recognize issues of scholarly ethics during the peer-review process | **SD** | **D** | **D/A=** | **A** | **SA** |
| 6. … discuss the mindset, social and ethical obligations required of a mentoring peer reviewer | **SD** | **D** | **D/A=** | **A** | **SA** |

**DIRECTIONS**: The peer review workshop was designed to prepare you for peer-reviewing manuscripts and abstract submissions. Please rate each of the program's components in terms of **preparing you to be a reviewer** by circling a response in the columns down the left hand side of the page. Next rate the quality of instruction (or materials) for the workshop's sub-components by circling a response in the **Quality of Instruction** columns down

the right-hand side of the page.

| **CONTRIBUTION TO PREP** | | | | | **COURSE COMPONENTS** | **QUALITY OF INSTRUCTION** | | | | |
| --- | --- | --- | --- | --- | --- | --- | --- | --- | --- | --- |
| **4=Great Value**  **3=Considerable Value**  **2=Moderate Value**  **1=Minimal Value**  **0=No Value** | | | | |  | **E=Excellent**  **G=Good**  **S=Satisfactory**  **M=Marginal**  **P= Poor** | | | | |
| **4** | **3** | **2** | **1** | **0** | 1. Session introduction | **E** | **G** | **S** | **M** | **P** |
| **4** | **3** | **2** | **1** | **0** | 2. Activity 1: Basic reviewer qualifications | **E** | **G** | **S** | **M** | **P** |
| **4** | **3** | **2** | **1** | **0** | 3. Activity 2: Good citizen reviewer approach | **E** | **G** | **S** | **M** | **P** |
| **4** | **3** | **2** | **1** | **0** | 4. Activity 3: Practice review activity | **E** | **G** | **S** | **M** | **P** |
| **4** | **3** | **2** | **1** | **0** | 5. Activity 4: Peer-review ethics | **E** | **G** | **S** | **M** | **P** |
| **4** | **3** | **2** | **1** | **0** | 6. Summary, wrap-up | **E** | **G** | **S** | **M** | **P** |

*Please continue on the back*

1. Given the amount of content covered and the activities, rate the length of the session. (Circle one)

**Too Short	Just Right	Too long**

2. What is the single best “thing” you learned from today’s workshop?

3. What should the presenters do differently next time the workshop is presented?

*Thank you for your feedback!*
