# Supplementary material for: Fundamentals of Scholarly Peer Review: A Workshop for Health Professions Educators on Practicing Scholarly Citizenship
Source: MedEdPORTAL. 2021 Aug 2;17:11174. doi: 10.15766/mep_2374-8265.11174 (PMC8335801; doi:10.15766/mep_2374-8265.11174)
Supplement: Supplementary file 1 — Peer Reviewer Training Presentation.pptxHandout Cover.pdfHandout and Activity Worksheets.docxFacilitator Guide.docxEvaluation.docx [file mep_2374-8265.11174-s001.zip › C. Handout and Activity Worksheets.docx]

**Appendix C. Fundamentals of Scholarly Peer Review Workshop – Participant Handout**

**Activity 1 Worksheet: Reflections on Reviewer Skills, Interest and Readiness**

**1.1. Check the topics for which you would be interested in reviewing or with which you have familiarity.**

|  | Administration of Educational Programs |  | Feedback |  | Principles of Scoring & Grading |
| --- | --- | --- | --- | --- | --- |
|  | Adult Learning |  | Flipped Classroom Methods (e.g., TBL or PBL) |  | Professionalism |
|  | Advanced Psychometrics (Classic, IRT, G-Theory) |  | Graduate Medical Education |  | Quality Improvement |
|  | Advising for career or specialty choice |  | Humanities |  | Remediation |
|  | Assessment |  | Human Subjects Research/IRB |  | Research Design-Qualitative |
|  | Asynchronous Learning |  | Informatics |  | Research Design-Quantitative |
|  | Bedside Teaching |  | Instruction/Teaching Strategies |  | Scholarship of Teaching & Learning |
|  | Clinical Reasoning |  | Inter-professional education |  | Selection & Recruitment |
|  | Competency-Based Education |  | Item Writing |  | Simulation |
|  | Continuing Medical Education |  | Large Group Instruction |  | Small group teaching methods |
|  | Curriculum Development |  | Leadership |  | Standard Setting |
|  | Data Management |  | Learning Community |  | Statistical Analysis |
|  | Diversity and Inclusion |  | Learning Theory |  | Survey Methods |
|  | Education Philosophy |  | MCQ Test Development |  | Systematic Review |
|  | Education Research |  | Mentoring, Counseling, Advising |  | Teamwork |
|  | Ethics |  | Meta-Analysis |  | Technology enhanced education |
|  | Evaluation |  | Methods of Scholarly Dissemination |  | Undergraduate Medical Education |
|  | Evaluation of Measures (Reliability & Validity) |  | Performance Assessment |  | Validity evidence |
|  | Faculty Development |  | Portfolios in Education |  | Wellness |

Other Topics, please list: _______________________________________________________________

____________________________________________________________________________________

____________________________________________________________________________________

**1.2. Please rate your skillset in each of the domains below, using this key:**

1= Very Unskilled (Novice, need constant help, support and feedback)

2= Unskilled (Beginning proficiency, but still need much guidance)

3= Intermediate Performer (Can do myself, with occasional guidance from an expert)

4= Skilled (Can perform autonomously, with a little help from a reference guide, the internet, or a guide)

5= Very Skilled (Expert, can teach and supervise others on this skill)

| **Rating** | **Domain** |
| --- | --- |
|  | Writing an abstract, report, or manuscript |
|  | Citing or referencing other authors |
|  | Providing constructive (formative) feedback |
|  | Summarizing the work/ideas/writing of someone else for an audience |
|  | Evaluating writing for structure, grammar, flow, voice |
|  | Statistical analysis and interpretation |
|  | Critically appraising qualitative research methods |

**1.3. What would you decide? (Circle your answer)**

Dear Dr. Shackleton:

Manuscript ID 22-095 entitled “Asteroids® and Electrocardiograms: Proof-of-Concept for Task-Switching

Simulation”^1^ has been submitted to our journal.

I invite you to review this manuscript. The abstract appears below. The time allotted for review is 14 days. Please let us know if you will accept this invitation to review. Click on the link below with your

decision.

**Yes…**I agree to review this manuscript

**No…**I will not review this manuscript

Sincerely,

Dr. Mohave Desert

Decision Editor, The Journal of Great Education

***************

BACKGROUND: Emergency medicine (EM) physicians are interrupted during patient care with such tasks as reading electrocardiograms (ECGs). This phenomenon is known as task-switching which may be a teachable skill.

OBJECTIVES: Our purpose was to evaluate the potential of a video game for simulating the cognitive demands required of task-switching.

CURRICULUM DESIGN: EM residents took a pretest on ECG interpretation and then a posttest while attending to a video game, Asteroids.®

IMPACT/EFFECTIVENESS: The 35 residents (63%) who participated, scored worse on the ECG posttest then they did on the pretest (t= 6.05, df=35, p<.001; effect size=1.14). There were no differences between genders or training level.

CONCLUSION: Interpreting ECGs while playing the Asteroids® game significantly lowered ECG interpretation scores. This shows the potential of this activity for training residents in task-switching ability. The next phase of research will test whether ECG reading performance while task-switching improves with practice.

**Fundamentals of Scholarly Peer Review Workshop**

**Activity 2 Worksheet: Evaluating the Helpfulness of Peer Reviewer’s Comments**

Below are 10 excerpts from reviews of various articles. Please rate the helpfulness of each excerpt as (1) Marginally Helpful, (2) Somewhat Helpful, or (3) Very Helpful to the authors’ quality improvement and to the journal editor’s decision making.

| **Comment** | **Rating** |
| --- | --- |
| Overall a very interesting read. A thorough proofread is required, preferably by a native English person. |  |
| While the manuscript is well structured, there are grammatical errors throughout that distract the reader from the important points. I encourage the authors to review the manuscript critically line by line, with a careful eye towards eliminating compound and passive-voice sentences, and those with dangling participles. Fancy words should be replaced by simpler ones. |  |
| The introduction sets the stage for the phenomenon being studied: how a transitional year affects professional identity development among medical trainees. The importance of understanding this phenomenon is made clear by noting that the transition can be stressful. At the end of the introduction there is a discussion of what the findings of study will add to what is known. I think it might be helpful to bring this earlier in the introduction, to make the case for this study earlier. |  |
| The literature on feedback in residency education is abundant; so much so that it is difficult to keep up with across various disciplines. There are numerous articles on constructive feedback published in a special edition of the Journal of Education Science. These are worth reading and may contribute to improving this paper. |  |
| “Qualitative methods” is a broad term. Could you please specify and give arguments for your methods of inquiry? In Figure 5, I read that you used thematic analysis, but you did not specify this in the text, nor reference it. |  |
| The authors do not describe the dyadic learning condition. However, the extensive literature on collaborative learning has demonstrated that, in order to observe the learning benefits of collaboration, it is not sufficient to place students into groups. Instead, tasks must be structured in way to induce positive interdependence among the members’ actions. The authors mention positive interdependence but have not seemed to grasp the importance of designing tasks such that this type interdependence arises during group work. I suggest that the authors describe the dyadic learning condition in more detail and explain why they expect positive interdependence to arise (or not). If not, I suggest that they do not call their intervention dyadic learning. |  |
| In the final paragraph of the results section, I wanted clarification of what "all instruction methods were believed to be valuable" referred to. What were the instructional methods other than hands on and didactics? Maybe list the other instructional methods referred to for clarification. |  |
| Regarding the results section, I was surprised to see such a limited section. In accordance to the design of the study and its complexity, this is really limited. |  |
| The research is presented in the standard form of scholarly writing. However, the authors have overlooked the inclusion of any study limitations. One that comes to mind is the lack of generalizability that occurs when conducting research within one program within one institution. |  |
| It would be useful to provide more detail about how the themes identified provide guidance in differentiating students. Please provide some specific hypothetical examples where these themes could be applied to both formative and summative feedback. |  |

**Fundamentals of Scholarly Peer Review Workshop**

**Activity 3 Worksheet: Practice Reviewing a Scholarly Sample and Writing Feedback Comments**

**Comparison of a Lecture to an Online Learning Module for Teaching Complex Clinical Subject Matter to Physicians-in-Training^[1]^**

**Abstract**

Introduction

Despite contemporary evidence that lectures rarely contribute to improvements in physician’s practice patterns, they remain the primary teaching method for educating resident physicians. Self-directed learning has been shown to be an effective alternative to lectures, at least among highly motivated adult learners. We hypothesized that an internet-based, independent instruction module containing complex information about the clinical diagnosis and management of Community-acquired Pneumonia (CAP) would lead to superior assimilation and retention of knowledge, when compared to a 40-minute content expert lecture on CAP.

Methods

A stratified random sampling procedure was used to assign internal medicine residents to educational treatment groups: Group A-didactic lecture and Group B computer-based instruction. Following instruction, each group took a 16-item multiple choice test, and a satisfaction survey. To assess knowledge retention, a second 16-item test was administered six weeks after the activities. Raw scores on the tests were converted to percentages. Scores were analyzed with a two-way analysis of variance with one repeated measure (Group x Time: Posttest-Delayed Posttest).

Results

Forty-six trainees, 23 in each group, completed the initial activity and test. The difference between scores was not statistically significant at either Time 1 (the immediate posttest), nor Time 2 (the six-week follow up). However, and interaction effect between Time and Group was observed (F=4.23; df=1, 23; *p≤*.05). Group A mean test scores (and standard deviation) seemed to have dropped at a faster rate from 51.3 (7.5) to 42.4 (7.7) over the six-week delay, while the drop in Group B mean test scores (and standard deviation) was more gradual from 49.6 (4.5) to 44.0 (4.9). Time spent on the learning activities, as well as satisfaction scores were not significantly different between the groups.

Conclusion

The significant interaction can be interpreted as a difference between the groups’ change in test performance over time. If we think of the first test score as how much was learned just after instruction and the second test score as how much learning was retained over six weeks, it appears that the two groups learned the same, but that the computer-based group retained more knowledge over time. Computer-based instruction may offer a distinct advantage in improving retention of knowledge concerning complex medical subject matter.

**Fundamentals of Scholarly Peer Review Workshop**

**Activity 4: Scholarship Case Studies: Reflection on Research and Reviewer Ethics**

Each of these brief scenarios describes common situations in research. Is there a problem? Write the

category of the “common” problem in the box next to the scenario.

| **Scenario** | **Problem Category** |
| --- | --- |
| 1. A team of faculty, residents and staff from Midwestern University formed the Medical Education Research Community of Practice (MER-COP) to work on education research projects. During their first year, they published a paper on the impact of a Team-Based Learning (TBL) preparation course on USMLE Step 1 scores. Later, the group submitted a sequel describing a similar study except this time the group reported the impact of TBL preparation course on USMLE Step 2 CK scores. |  |
|  |  |
| 2. A medical student, Belinda Charm, took time off from medical school to complete a public health degree. With her extra time, she volunteered to prepare and teach a lesson to medical students on using ultrasound to evaluate the thyroid for pathology. The voluntary lesson (extracurricular) was inserted into the month-long endocrine system curriculum block, which concluded with a written examination. She became aware that the students who participated in her lesson performed better on the written exam than those who did not. Excited to share her discovery, she submitted a poster abstract to a regional conference to share her teaching methods. |  |
|  |  |
| 3. Reza D. Rufe, PhD is a medical educator who teaches the biopsychosocial approach to health, illness and healthcare delivery to first-year medical students. She is also the reviewer for a prestigious medical education journal. She is enthusiastic about a manuscript she just reviewed because the findings reinforced the value of her topic to medical education. To motivate her learners about this topic, and because she was sure this manuscript would eventually be published, she included the manuscript’s results in one of her lectures. |  |
|  |  |
| 4. A well-meaning resident, Milton Frost, jumped in to help with a research question generated by his peer. The two designed an experiment to study whether an online learning module with well-placed multiple-choice questions was superior to a lecture format for teaching about aspiration pneumonia. Milton volunteered to perform a literature review and write the first draft of the manuscript’s introduction. He carefully referenced articles with titles that fit with the content of the paper and then turned it over to his co-authors before graduating. The coauthors were surprised when their well-written manuscript was rejected. The primary reason for the rejection had to do with the citations. |  |
|  |  |
| 5. Dr. GW Crile is a veteran researcher who has been in medical education for over 30 years. She is a prolific author, with nearly 150 publications, and serves as a reviewer for a high-impact journal. She has been mentoring a junior faculty member, Dr. Bunts, who has just gotten started in the field. She was excited to be asked to review a manuscript written by her protégé. She wants to be sure that Dr. Bunts gets a fair shake, and she was not directly involved in the project presented in his manuscript, so she agrees to do the review. |  |

**Fundamentals of Scholarly Peer Review Workshop**

**Workshop Wrap-up: Where to go on your path to becoming a good reviewer.**

1.	Before accepting, decide whether you have time and sufficient interest in the topic to perform the review.

2.	Become thoroughly familiar with the submission venue (journal, conference, or grant): the authors’ purpose, the body of literature that the authors cite, the research question, the research methods, data analysis and reporting of results, and contents of the discussion.

3.	Frame your review as if you are communicating to the editor. Start with a brief summary of the study. Provide strengths as well as shortcomings. Address the relevance of the research question, the originality of the research, the fit between methods and research question, overall presentation…including statements about writing style, grammar, and spelling, etc. Determine whether the authors recognized and addressed the appropriate limitations and whether the discussion was framed appropriately without going beyond the study’s results.

4.	If there is a fatal flaw in the manuscript, be clear but not offensive as to the problem. Provide detailed comments regarding the flaw, such as: a mismatch between question and methods. Provide suggestions as to what the authors need to do in order to “fix” the problem.

5.	Outline your comments similarly to the outline of the submission (Introduction, Methods, Results, Discussion, and Conclusion). Number your comments and label them as either “major” criticisms or “minor” criticisms.

6.	Have a growth rather than fixed mindset. Nurture the authors to improve.

7.	Reviewers may suggest sources of confusion in the writing but should not take it upon themselves to edit the paper.

8.	If you believe that the submission involves a violation of scholarly ethics, be sure to call your concern to the attention of the editor.

**Fundamentals of Scholarly Peer Review Workshop**

**References and Resources**

1.	Academic Medicine Staff. AM Rounds-Beyond the pages of Academic Medicine: Peer Reviewer Resources. <http://academicmedicineblog.org/category/peer-reviewer-resources/>. Accessed January 14, 2019.

2.	Artino AR, Driessen EW, Maggio LA. Ethical shades of gray: International frequency of scientific misconduct and questionable research practices in health professions education. Acad Med. 2019;94(1):76-84.

3.	Azer SA, Ramani S, Peterson R. Becoming a peer reviewer to medical education journals. Med Teach. 2012;34(9):698-704.

4.	Aziz F, Yeh B, Emerson G, Way DP, San Miguel C, King AM. Asteroids® and Electrocardiograms: Proof of Concept of a Simulation for Task-Switching Training. West J Emerg Med. 2019 Jan;20(1):94-7. [Used with permission of the authors].

5.	Berry A, Coplit L, Fornari A, Greenberg L, Mezger K, Pasquale S, Shapiro J, Willett L, Vasan N, et al. Annotated bibliography of journal for educational scholarship. Washington, DC; Association of American Medical Colleges Group on Educational Affairs. Revised July, 2019. https://www.aamc.org/media/38166/download.

6.	Bierer B, Foshee C, Uijtdehaage S. Strategies to remain current with the medical education field. MEd Sci Educ. 2015;25:163-70.

7.	Bordage G, Caelleigh A. Special edition on review criteria for research manuscripts. Acad Med. 2001;76:863-975. https://www.aamc.org/members/gea/167684/guide_for_reviewers.html.

8.	Bourne PE, Korngreen A. Ten simple rules for reviewers. PLoS Comput Biol. 2006 Sep;2(9):e110.

9.	Colliver JA. Commentary on “Review Criteria for Research Manuscripts.” Teach Learn Med 2002;14(2):75-6.

10.	Crandall S, O’Sullivan P. Scholarly Writing for Medical Education Research. Medical Education Research Certificate (MERC) Workshop. Washington, DC; Association of American Medical Colleges.

11.	Cummings M. The hidden mentor: the role of the reviewer. In AM Rounds-Beyond the pages of Academic Medicine: Peer Reviewer Resources. <https://academicmedicineblog.org/for-reviewers-series-the-hidden-mentor-the-role-of-the-reviewer/>. Published September 26, 2013. Accessed December 16, 2020.

12.	Dialogue. Image obtained from Gerd Altmann, Pixabay. Free for commercial use, no attribution required. <https://pixabay.com/illustrations/dialogue-system-web-news-personal-2546121/>

13.	Dudek NL, Marks MB, Wood TJ, Lee AC. Assessing the quality of supervisor’s completed clinical evaluation reports. Med Educ 2008; 42:816-22.

14.	Durning SJ, Carline JD, eds. Review Criteria for Research Manuscripts, 2nd ed. Washington, DC: Association of American Medical Colleges; August, 2015. <https://store.aamc.org/>

15.	Eva KW. The reviewer is always right: peer review of research in Medical Education. Med Educ. 2009;43(1):2-4.

16.	Eva, KW. How would you like your salami? A guide to slicing. Med Educ 2017; 51: 456-7.

17.	Foreman H. How to review manuscripts-your ultimate checklist. Philadelphia, PA; Elsevier. August 28, 2015. <https://www.elsevier.com/connect/how-to-review-manuscripts-your-ultimate-checklist>

18.	Hall GM. How to write a paper. 5th ed. Hoboken, NJ; John Wiley & Sons, Ltd. 2013.

19.	ICMJE Form for disclosure of potential conflicts of interest. International committee of medical journal editors. December, 2019. http://www.icmje.org/

20.	Karlin ES. Chapter 23: Reviewer’s etiquette. In Durning SJ, Carline JD, eds. Review Criteria for Research Manuscripts, 2nd ed. Washington, DC: Association of American Medical Colleges; August 2015:197-202.

21.	MacAuley D. Chapter 16: The role of the manuscript assessor. In Hall GM, ed. How to write a paper. 5th ed. Hoboken, NJ; John Wiley & Sons, Ltd. 2013. 102-14.

22.	Maggio LA, Artino AR, Picho K, Driessen EW. Are you sure you want to do that? Fostering the responsible conduct of medical education research. Acad Med. 2018;93(4):544-9.

23.	Magnifying Glass Clipart on Workshop Handbook cover and Slides 1-11 of Powerpoint Slides. Image by unknown, retrieved from: <http://res.publicdomainfiles.com/pdf_view/60/13534575618789.png> on February 1, 2021. Image is in the public domain.

24.	Mavis B, Durning SG, [Uijtdehaage S](https://www-ncbi-nlm-nih-gov.ccmain.ohionet.org/pubmed/?term=Uijtdehaage%20S%5BAuthor%5D&cauthor=true&cauthor_uid=30556420). Authorship order in medical education publications: In search of practical guidance for the community. Teach Learn Med. 2019;31(3):288-97.

25.	Pechenik JA. "Writing Summaries and Critiques." In Gilpin R., ed. A Short Guide to Writing about Biology. 6th ed. New York, NY; Pearson: 2007. 130-8.

26.	Recommendations for the conduct, reporting, editing, and publication of scholarly work in medical journals. International committee of medical journal editors. December, 2019. http://www.icmje.org/

27.	Reporting guidelines for main study types. Equator Network. University of Oxford; UK EQUATOR Centre. December 28, 2020. <https://www.equator-network.org/>

28.	Reviewer Team Around a Table Clipart on Slide 5 of Powerpoint. Image by Augusto Ordonez, retrieved from Pixabay: <https://pixabay.com/vectors/meeting-table-work-team-4386216/> on January 31, 2021. Free for commercial use, no attribution required.

29.	Richards BF, Cardell EM, Chow CJ, Moore KB, Moorman KL, O’Connor M, Hart SE. Discovering the benefits of group peer review of submitted manuscripts. Teach Learn Med. 2020 Jan 1;32(1):104-9.

30.	Roberts LW, Coverdale J, Edenharder K, Louie A. How to Review a Manuscript: A “Down-to-Earth” Approach. Acad Psychiatry. 2004 Summer;28(2):81-87.

31.	Spigt M, Arts IC. How to review a manuscript. J Clin Epidemiol. 2010;63:1385–90.

32.	Step by Step guide to reviewing a manuscript. Hoboken, NJ; John Wiley & Sons, inc. <https://authorservices.wiley.com/Reviewers/journal-reviewers/how-to-perform-a-peer-review/step-by-step-guide-to-reviewing-a-manuscript.html>

33.	Sullivan SE, Baruch Y, Schepmyer H. The why, what, and how of reviewer education: A human capital approach. J Manage Educ. 2010;34(3):393–429.

34.	Tolsgaard MG, Ellaway R, Woods N, Norman G. Salami‑slicing and plagiarism: How should we respond? Adv Health Sci Educ. 2019;24:3–14.

35.	Uijtdehaage S, Mavis B., Durning SJ. Whose paper is it anyway? Authorship criteria according to established scholars in health professions education. Acad Med. 2018;93(8):1171-5.

36.	Writing, Quill, Books Clipart on Workshop Handbook cover and Slides 1-11 of Powerpoint Slides. Image by Janet Gooch, Retrieved from Pixabay: <https://cdn.pixabay.com/photo/2015/11/14/20/26/writing-1043622_960_720.png>. Free for commercial use, no attribution required.

1. The workshop authors would like to thank Jerome E. Stasek, MD; The Ohio State University College of Medicine, Associate Professor of Pulmonary, Critical Care, and Sleep Medicine for permission to use this case as an example.
